# Near-Infrared Fluorescence Imaging of Pancreatic Cancer Using a Fluorescently Labelled Anti-CEA Nanobody Probe: A Preclinical Study

**DOI:** 10.3390/biom13040618

**Published:** 2023-03-30

**Authors:** Labrinus van Manen, Lizzie D. A. N. de Muynck, Victor M. Baart, Shadhvi Bhairosingh, Pieterjan Debie, Alexander L. Vahrmeijer, Sophie Hernot, J. Sven D. Mieog

**Affiliations:** 1 Department of Surgery, Leiden University Medical Center, 2333 ZA Leiden, The Netherlands; 2 Laboratory for In Vivo Cellular and Molecular Imaging (ICMI-MIMA/BEFY), Vrije Universiteit Brussel, 1050 Brussels, Belgium

**Keywords:** pancreatic cancer, pancreatic ductal adenocarcinoma, nanobody, antibody, fluorescence, near-infrared, surgery, zwitterionic

## Abstract

Molecular fluorescence-guided surgery using near-infrared light has the potential to improve the rate of complete resection of cancer. Typically, monoclonal antibodies are being used as targeting moieties, however smaller fragments, such as single-domain antibodies (i.e., Nanobodies^®^) improve tumor specificity and enable tracer injection on the same day as surgery. In this study, the feasibility of a carcinoembryonic antigen-targeting Nanobody (NbCEA5) conjugated to two zwitterionic dyes (ZW800-1 Forte [ZW800F] and ZW800-1) for visualization of pancreatic ductal adenocarcinoma (PDAC) was investigated. After site-specific conjugation of NbCEA5 to the zwitterionic dyes, binding specificity was evaluated on human PDAC cell lines with flow cytometry. A dose escalation study was performed for both NbCEA5-ZW800F and NbCEA5-ZW800-1 in mice with subcutaneously implanted pancreatic tumors. Fluorescence imaging was performed up to 24 h after intravenous injection. Furthermore, the optimal dose for NbCEA5-ZW800-1 was injected in mice with orthotopically implanted pancreatic tumors. A dose-escalation study showed superior mean fluorescence intensities for NbCEA5-ZW800-1 compared to NbCEA5-ZW800F. In the orthotopic tumor models, NbCEA5-ZW800-1 accumulated specifically in pancreatic tumors with a mean in vivo tumor-to-background ratio of 2.4 (SD = 0.23). This study demonstrated the feasibility and potential advantages of using a CEA-targeted Nanobody conjugated to ZW800-1 for intraoperative PDAC imaging.

## 1. Introduction

Molecular fluorescence-guided cancer surgery using near-infrared (NIR) light is an innovative and promising optical imaging technique that entered the surgical theatre in the past years [1]. It needs a camera system that is able to excite an (intravenously injected) fluorophore conjugated to a tumor-specific targeting molecule and detect its emitted fluorescence [2]. The technique is thought to be of significant added value to improve intraoperative navigation, which could be especially relevant during pancreatic cancer surgery. Pancreatic cancer has a dismal prognosis as most of the patients (around 80%, [3]) have either locally advanced or metastasized disease at diagnosis. During surgery, margin assessment is of utmost importance since tumor-positive resection margins are encountered in up to 70% of the cases [4,5]. Distinguishing tumor tissue from benign surrounding tissues using an imaging technique that is easy to use, such as NIR fluorescence imaging, is one of the key elements to improve the quality of pancreatic surgery [6] of the most commonly targeted and studied biomarkers for pancreatic cancer is the carcinoembryonic antigen (CEA), which is a glycoprotein involved in cell adhesion and is overexpressed in more than 90% of pancreatic cancers [7,8,9]. CEA is still overexpressed in tumor tissue after neoadjuvant therapy, which makes this an excellent target moiety, and therefore an interesting candidate for targeted fluorescence imaging, since more patients are being treated with neoadjuvant therapy followed by surgery and/or adjuvant chemotherapy [10]. It has already been demonstrated that an anti-CEA monoclonal antibody conjugated to a 700 nm fluorophore (SGM-101) could clearly visualize pancreatic tumors during surgery [11]. Nonetheless, monoclonal antibodies typically limit NIR fluorescence imaging due to restricted tumor penetration and long blood circulation time, resulting in suboptimal pharmacokinetics, low signal-to-noise ratios, and extended time between injection and surgery (3–5 days). Single domain antibodies or Nanobodies^®^, which are the smallest (15 kDa) intact antigen-binding fragments derived from camelid heavy-chain only antibodies, are excellent candidates for molecular imaging as they show rapid target recognition and clearing from the circulation via renal excretion [12]. This results in lower background fluorescence, improved tumor specificity, and enables tracer injection just prior to surgery [13,14,15,16].

In addition to Methylene Blue and Indocyanine Green, during the past years, many NIR fluorophores have become available for conjugation to target moieties, of which IRDye800CW is the most commonly used NIR fluorophore [17]. A fundamental problem of these conventional NIR fluorophores for tumor targeting is that they are polysulfonatic and highly anionic, resulting in non-specific uptake in tissue and organs, leading to higher background fluorescence, which could be clinically relevant for the implementation of fluorescence-guided pancreatic surgery since both the pancreatic head is anatomically closely related to the liver and pancreatic tumors often metastasize to the liver. To overcome this limitation, a new chemical class of fluorophores, so-called zwitterionic dyes, was developed. Having strong charges (sulfonates and quaternary amines) that are balanced electrically and geometrically over the surface of the molecule, zwitterionic NIR fluorophores are self-shielding and exhibit extremely low non-specific binding and tissue uptake in vivo after intravenous injection [18].

In this study, the main goal was to assess the feasibility of a CEA-targeted Nanobody conjugated to two zwitterionic dyes, ZW800-Forte and ZW800-1, to facilitate the visualization of pancreatic cancer and characterize the results with respect to tumor specificity, mean fluorescence intensity and tumor-to-background ratio.

## 2. Materials and Methods

### 2.1. Fluorescent Tracers

Two fluorescent zwitterionic dyes (ZW800-1 Forte (ZW800F) and ZW800-1), activated with maleimide for thiol-linkage, were conjugated site-specifically to either a CEA-targeted Nanobody (NbCEA5) or a control Nanobody (R3B23) [15,19]. Both Nanobodies were generated in the context of previous studies and produced as described previously [14]. ZW800-F has two absorption peaks, at 685 nm and 754 nm, respectively, and an emission peak of 772 nm in phosphate-buffered saline (PBS) [20]. ZW800-1-Maleimide has an absorption peak of approximately 772 nm and an emission peak of 788 nm in PBS [20].

In brief, the Nanobodies were genetically engineered to carry a carboxy-terminal tag consisting of a hexahistidine-tag, a 14 amino acid linker, and a cysteine-tag, and were subsequently expressed in Escherichia coli cultures [19]. Purification of the Nanobodies from periplasmic extracts was performed by immobilized metal affinity chromatography, after which size-exclusion chromatography (SEC) was applied [21]. Prior to conjugation to the dyes, Nanobodies were reduced by incubation at 37 °C with 180× molar excess of 2-MEA (ACROS organics, Thermo Fisher Scientific, Rockford, IL, USA). After 90 min, 2-MEA was removed by buffer exchange on a PD-10 desalting column (GE Healthcare, Chicago, IL, USA) using PBS (pH 7.4) as an elution buffer. The reduced Nanobody was then incubated with a 5-fold molar excess of either ZW800F or ZW800-1, dissolved at 20 mg/mL in Dimethyl sulfoxide. SEC was used to purify the fluorescently labeled Nanobodies (Superdex 75 10/300 GL, PBS as running buffer at 1.0 mL/min) as well as to assess purity afterwards. The concentration of the conjugated Nanobodies was determined via spectrophotometry (NanoDropTM 2000, Thermo Fisher Scientific, Rockford, IL, USA) and the degree of labeling, calculated as the ratio of the dye concentration and protein concentration, taking a correction factor of 3% into account for the absorbance of the dye at 280 nm [22].

### 2.2. Human Cancer Cell Lines

Two pancreatic cancer cell lines were used for this experiment. BxPC3-luc2 cells were bought by Perkin Elmer (Waltham, MA, USA) and PANC-1 were obtained from ATCC. BxPC-3-luc2 cells were cultured in RPMI 1640 cell culture medium (Gibco, Invitrogen, Carlsbad, CA, USA) and PANC-1 cells were cultured in Dulbecco’s Modified Eagle Medium (DMEM) + GlutaMAX™ cell culture medium (Gibco, Invitrogen, Carlsbad, CA, USA). l-glutamine, 25 mM HEPES, 10% fetal bovine serum (FBS; Hyclone, Thermo Scientific, Rockford, IL, USA), and penicillin/streptomycin (both 100 IU/mL; Invitrogen) were added as supplements [23]. All cell lines were cultured in absence of mycoplasma, which was confirmed using a polymerase chain reaction. The cells were grown in a humidified incubator (37 °C, 5% CO_2_) to 90% confluence, and were detached afterwards with trypsin/EDTA [23]. Viability was assessed using Trypan Blue staining in 0.4% solution (Gibco, Invitrogen).

### 2.3. Flow Cytometry

After detachment and assessment of the viability of the cell lines, 0.5 × 106 cells were resuspended per tube in ice cold PBS. Hereafter, cells were washed twice in ice-cold PBS supplemented with 0.5% bovine serum albumin (BSA) and incubated with the anti-CEA monoclonal antibody labeled by APC (FAB41281A-APC; R&D Systems, Minneapolis, MN, USA), NbCEA5-ZW800F, NbCEA5-ZW800-1 or R3B23-ZW800-1 for 30 min on ice (concentrations: 10 nM, 100 nM, and 1000 nM). After washing twice, cells were resuspended in 400 μL PBS/0.5%BSA and analyzed on an LSRII flow cytometer (Biosciences, Franklin Lanes, Evansville, IN, USA) using the 670/14 laser for measuring APC signals and 780/60 laser for measuring ZW800-1 or ZW800F signals [23,24]. All steps were performed to avoid exposure to light. Histograms were generated using FlowJo software (TreeStar, Ashland, OR, USA, version 10.6.2).

### 2.4. Animal Procedures

The Animal Welfare Committee of Leiden University Medical Center approved all animal experiments for animal health, ethics, and research. Six-week-old athymic female mice (CD1-Foxn1nu, Charles River Laboratories, Wilmington, MA, USA) weighing between 20 and 35 g were housed in ventilated cages. Normal pellet food (as NIR fluorescence imaging was performed in the 800 nm range) and sterilized water were provided ad libitum. Throughout tumor inoculation and imaging procedures, animals were anesthetized with 4% isoflurane for induction and 2% isoflurane for maintenance. To induce subcutaneous tumors, BxPC3-luc2 cells (Perkin Elmer, Waltham, MA, USA) were injected at either one or two sites on the back of the mice (500,000 cells per spot). The pancreatic orthotopic models were performed according to Moreno et al. [25]. In brief, after performing a lateral incision in the left flank, the pancreas was exposed, and consequently, 500,000 BxPC3-luc2 cells (resuspended in 50 μL PBS) were injected into the body of the pancreas. All mice undergoing orthotopic tumor implantation received painkilling (0.1 mg/kg buprenorphine subcutaneously, every 8 h) during and after the surgical procedure as needed. Following imaging, the mice were euthanized using carbon dioxide, and organs were collected and imaged for fluorescence to evaluate the biodistribution pattern of the tracers.

### 2.5. Camera Systems

The Pearl Impulse whole animal imaging system (LI-COR Biosciences, Lincoln, NE, USA) and the Quest Artemis (Quest Medical Imaging, Middenmeer, The Netherlands) open fluorescence camera system were used for fluorescence measurements. The excitation wavelengths for the Pearl Impulse imager and Quest Artemis imaging systems are 685/785 nm and 680/793 nm, respectively, and the collected emission wavelengths were 720/820 nm and 700–800/805–850 nm, respectively.

### 2.6. Experimental and Imaging Procedure

The subcutaneously implanted tumors were monitored using a digital caliper and mice were considered for imaging if the tumor size was above 5 by 5 mm. A dose escalation study was performed for both tracers, varying from 0.5, 1, 2, and 4 nmol based on the fluorophore concentration (N = 3 mice/group, except for the 4 nmol group: N = 2). For the subcutaneous models imaging was performed 1, 2, 4, 8, and 24 h after intravenous injection. The orthotopic implanted tumor models (N = 2) were imaged 2 h after intravenous injection. Biodistribution was performed in the orthotopic tumor models immediately after the euthanasia of the animals.

### 2.7. Ex Vivo Analysis

After imaging, the excised tumors were fixed in 4% formalin and embedded in paraffin (FFPE) blocks. All tissues were sectioned, and consequently, fluorescence imaging at 800 nm was performed on the slides using the Odyssey imager (LI-COR Biosciences, Lincoln, NE, USA) and AxioZ1 scanner (Carl Zeiss AG, Oberkochen, Germany). Additionally, hematoxylin-eosin (HE) staining, CEA-specific staining, DAPI for routine nuclear staining, and staining with a negative mouse antibody was performed as previously described [24], and consequently, these images were digitalized using the 3D HISTECH Confocal imager (Sysmex, Etten-Leur, The Netherlands).

### 2.8. Statistical Analysis

Statistical analysis and generation of graphs were performed using GraphPad Prism software (version 8, GraphPad Software Inc., La Jolla, CA, USA). Tumor-to-background ratios (TBRs) were calculated by dividing the mean fluorescence intensity (MFI) of the tumor (in arbitrary units (AU)) by the signal from the surrounding tissue (orthotopic model: adjacent normal pancreas; subcutaneous model: adjacent abdominal background signal in donut shape surrounding the tumor) and were reported as mean plus standard deviation. The main outcomes (MFI and TBR) were compared using an independent sample T-test and one-way ANOVA taking multiple comparisons into account. The *p*-values of < 0.05 were considered to be statistically significant.

## 3. Results

### 3.1. Conjugation and Binding Specificity

NbCEA5 and R3B23 were successfully conjugated with ZW800F and ZW800-1 and eluted as a single peak on analytical SEC. The degree of labeling ranged between 0.8 and 1.0. NbCEA5-ZW800F binding to living tumor cells was evaluated using flow cytometry in BxPC3-luc2 cells. A right shift was noticed for NbCEA5-ZW800F compared to R3B23-ZW800F (negative control Nanobody). Furthermore, NbCEA5-ZW800-1 was evaluated in the high CEA positive BxPC3-luc2 and the modest CEA positive PANC-1 cell line, demonstrating a larger right shift for the BxPC3-luc2 cells and thereby confirming specific binding (Supplementary data Figure S1).

### 3.2. In Vivo NIR Fluorescence Imaging of Pancreatic Tumors

**Figure 1:**
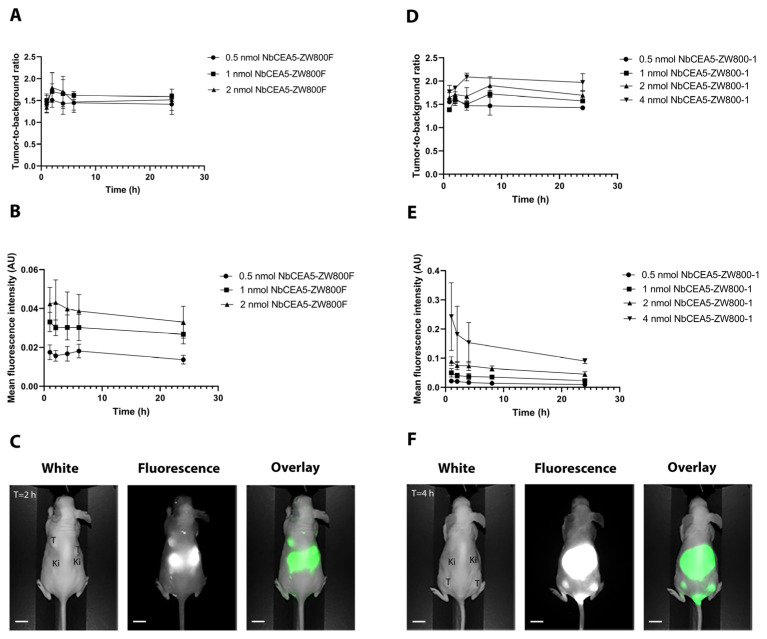
Left panel: Average tumor-to-background ratios (**A**), mean fluorescence intensities in the tumor (**B**), and representative images of the optimal doses based on the TBR acquired by the Pearl Imager (**C**) for NbCEA5-ZW800F. Right panel: Average tumor-to-background ratios (**D**), mean fluorescence intensities in the tumor (**E**), and representative images of the optimal doses based on the TBR acquired by the Pearl Imager (**F**) for NbCEA5-ZW800-1. Scale bars are 10 mm. Abbreviations: T = tumor; Ki = kidneys.

In vivo NIR fluorescence imaging using NbCEA5-ZW800F was performed over a 24 h period, resulting in a maximal TBR at 2 h after injection. The mean TBRs of 1.5 (SD = 0.17), 1.7 (SD = 0.18), and 1.8 (SD = 0.34) were observed for the 0.5, 1, and 2 nmol dose groups, respectively. MFIs for all time points in all dose groups were below 0.10 AU, and therefore the intrinsic fluorescence uptake of NbCEA5-ZW800F was considered to be not sufficient to proceed in further experiments. A dose-escalation study using NbCEA5-ZW800-1 was then performed in 4 dose groups (0.5 nmol; 1 nmol; 2 nmol; 4 nmol) demonstrating a significantly higher TBR for the 4 nmol dose group at 4 h after injection compared to the lower dose groups (mean TBR 2.1 [SD = 0.09]; *p* = 0.009). Furthermore, MFIs of the 4 nmol dose group were above or around 0.10 AU for all imaging time points (Figure 1).

**Figure 2:**
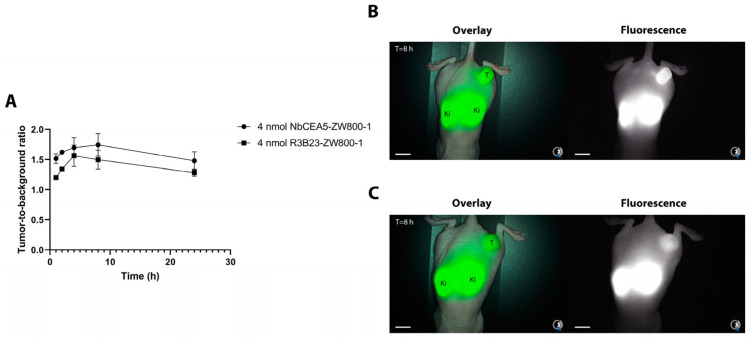
Comparison of tumor-to-background ratios over time for both NbCEA5-ZW800-1 and NbR3B23-ZW800-1 (control Nanobody) (**A**). Representative fluorescence overlay image acquired by the Quest Imaging system for NbCEA5-ZW800-1 (**B**) and NbR3B23-ZW800-1 (**C**) at the optimal imaging time point (8 h). Scale bars are 10 mm. Abbreviations: T = tumor; Ki = kidneys.

**Figure 3:**
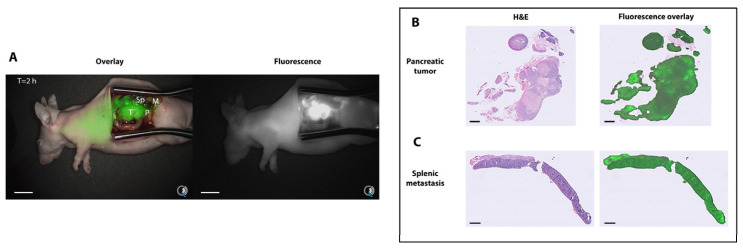
Fluorescence overlay image of an orthotopic implanted pancreatic tumor model including splenic metastases 2 h after injection of 4 nmol NbCEA5-ZW800-1 (**A**). Scale bars are 10 mm. Note: the kidneys were removed. The presence of tumor cells was histologically confirmed in (**B**,**C**). Scale bars are 1000 µm. Abbreviations: T = tumor; Sp: spleen; M = metastasis; P = normal pancreas.

Next, NIRF imaging of subcutaneous tumors after injection of both NbCEA5-ZW800-1 (N = 6) and R3B23-ZW800-1 (N = 3) was performed using the Quest Artemis clinical camera system. At 8 h after injection, the mean TBR was 1.8 (SD = 0.19) for the NbCEA5-ZW800-1 and 1.5 (SD = 0.16) for R3B23-ZW800-1, which was, however, not statistically significantly different (*p* = 0.09; Figure 2). In the orthotopic tumor models (N = 2), fluorescence clearly accumulated 2 h after injection of NbCEA5-ZW800-1 in the pancreatic tumors with a mean TBR of 2.4 (SD = 0.23; Figure 3). In one mouse, two splenic metastases could also be visualized with a mean TBR of 1.8 (SD = 0.42).

### 3.3. Biodistribution and Histologic Analysis

**Figure 4:**
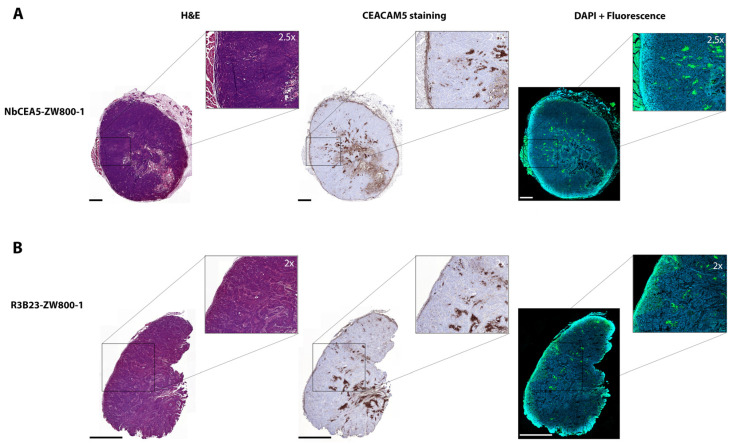
Representative examples of hematoxylin-eosin (HE) staining, CEACAM5 staining, and combined DAPI staining with NIR fluorescence (800 nm) on ex vivo tumor tissue sections of mice injected with NbCEA5-ZW800-1. (**A**) Representative examples of hematoxylin-eosin (HE) staining, CEACAM5 staining, and combined DAPI staining with NIR fluorescence (800 nm) on ex vivo tumor tissue sections of mice injected with NbR3B23-ZW800-1. (**B**). Scale bars are 1000 µm.

The presence of tumor cells for all detected fluorescent lesions was histologically confirmed. Ex vivo histological analysis showed that NbCEA5-ZW800-1 is fully penetrating the tumors and selectively targeting the CEA-positive tumor cells, whereas R3B23-ZW800-1 is not. The microscopic fluorescence signals of the NbCEA5-ZW800-1 are correlated with the CEACAM5 expression. (Figure 4). Biodistribution values for the relevant organs showed an MFI of 4.63 (SD = 0.47) for the kidney, 0.13 (SD = 0.03) for the tumor, 0.11 (SD = 0.04) for the liver, 0.05 (SD = 0.005) for the pancreas, and 0.02 (SD = 0.003) for muscle. Relevant tumor-to-organ ratios were 1.2 (tumor-to-liver); 7.9 (tumor-to-muscle), 2.6 (tumor-to-pancreas), and 0.03 (tumor-to-kidney, Supplementary data Figure S2).

## 4. Discussion

In this preclinical study, a CEA-targeted Nanobody, conjugated to zwitterionic dyes from the ZW800 family, was evaluated for intraoperative visualization of pancreatic tumors. Maleimide conjugation of this Nanobody to the zwitterionic dye ZW800-1 (NbCEA5-ZW800-1) showed superior tumor accumulation in terms of MFI and TBR compared to conjugation to the zwitterionic dye ZW800F. Furthermore, by using NbCEA5-ZW800-1, both subcutaneously and orthotopically implanted tumors could be visualized early (from 2 h) after tracer injection. Tumor-specific uptake was confirmed histologically. The results could indicate that the designed tracer has the potential of being translated for in-human use.

This CEA-targeting Nanobody was designed previously for clinical utility in both nuclear and fluorescence imaging. However, until now it has not been clinically translated [19]. Recently, this Nanobody has been demonstrated to visualize colorectal and pancreatic cancer in preclinical studies, although it was conjugated with another fluorescent dye, i.e., IRDye800CW [14,15,16]. For the pancreatic orthotopic model, it showed similar results concerning TBR values compared to our results (2.7 vs. 2.4), however, biodistribution patterns were not described in these studies [14]. Furthermore, the TBR values of these studies were difficult to compare as the TBR was determined on images taken from a different camera system (Maestro CRI imaging system) with different excitation and emission filters.

The designed tracer NbCEA5-ZW800-1 has the advantage of being mainly renally cleared and also demonstrated early tumor visualization after intravenous injection. An advantage of using Nanobodies compared to monoclonal antibodies is shorter imaging times although with lower TBRs and MFIs, which was in concordance with the results of Baart et al. [26]. In that study, uPAR-targeted Fab- (50–55 kDa) and Fab 2 (100–110 kDa) fragments were compared to their humanized parental antibody, thereby showing a greatly improved time-to-imaging, while the antibody itself demonstrated a superior peak fluorescence.

The strong kidney accumulation is one of the drawbacks of Nanobody-based tracers in general, however, it has to be expected that the kidney signal will not overwhelm the tumor signal in humans, since the kidneys are located retroperitoneally and are surrounded by perinephric fat. Future studies can directly compare this Nanobody construct to either a ZW800-1 labeled monoclonal anti-CEA antibody or conjugation of this Nanobody to other NIR fluorophores, or both, with respect to the tumor specificity and biodistribution patterns. Biodistribution studies have been performed for an anti-CEA targeted monoclonal antibody labeled to a 700-nm fluorophore, SGM-101, although only using Single Photon Emission Computed Tomography-Computed Tomography (SPECT-CT) imaging, making a fair comparison of our results to their tumor to organ ratios difficult, however, tracer accumulation in the kidneys seemed to be much higher for the monoclonal antibody compared to the Nanobody [27]. Future studies should therefore focus on biodistribution patterns of a monoclonal antibody versus a Nanobody labeled to ZW800-1 to evaluate the tumor-to-organ ratios (especially liver tissue), which would be clinically relevant since both the pancreatic head is anatomically close to the liver and pancreatic tumors often metastasize to the liver.

The results of this study could implicate that this novel anti-CEA Nanobody, NbCEA5-ZW800-1, has the potential to be translated into the clinic. As CEA is also overexpressed in other cancers, such as colorectal cancer, this tracer could also be used for intraoperative fluorescence imaging or endoscopic fluorescence imaging of other cancers [28]. It would also be useful to perform nuclear labeling, enabling nuclear imaging using for instance Positron Emission Tomography-Computed Tomography (PET-CT). An advantage of using CEA as a targeting moiety is that the CEA expression in PDAC is minimally affected by neoadjuvant chemoradiotherapy, making this tracer suitable for both intraoperative optical imaging and preoperative staging using PET-CT [10,29]. Patient selection could be performed based on elevated CEA levels as these are correlated to CEA expression profiles [30]. Future studies should focus on the optical characteristics and the effect on biodistribution of conjugation of this Nanobody to other NIR fluorophores. Based on these results the design of this CEA-targeted Nanobody tracer could be further optimized. Furthermore, the search for NIR fluorophores should not be limited to NIR-I fluorophores, as recently also organic fluorophores that can be used for conjugation and able to be detected in the NIR-II window (1000–1400 nm) have been developed [31]. The advantages of imaging in the NIR-II window are weaker autofluorescence, lower absorption, and reduced scattering, thereby improving the penetration depth and image quality, which has recently been demonstrated in humans during liver surgery [32].

## 5. Conclusions

In conclusion, this study demonstrates the feasibility of a novel fluorescent tracer, NbCEA5-ZW800-1, for intraoperative pancreatic cancer imaging in a preclinical animal study, as it showed tumor-specific fluorescence uptake and a short time to image. It could indicate that the designed tracer has the potential of being translated for clinical implementation in order to reduce the percentage of tumor-positive resection margins during pancreatic cancer surgery, although future studies should focus on the comparison of conjugation strategies to other NIR fluorophores, thereby optimizing the design of this CEA targeted Nanobody tracer.

## Data Availability

All data are available as requested by the corresponding authors.

## Supplementary Materials

The following supporting information can be downloaded at: https://www.mdpi.com/article/10.3390/biom13040618/s1, Figure S1: Overview of flowcytometry results. Figure S2: Biodistribution data of NbCEA5-ZW800-1 in the pancreatic orthotopic models (N = 2). The scale bar is 10 mm. Abbreviations: Lu = lungs; Ht = heart; Li = liver; Tu = tumor; Pa = pancreas; Sp = spleen; Int = small intestines; St = stomach; Ki = kidneys; Co = colon; Sk = skin; Mu = muscle.
