# Supplementary material for: Near-Infrared Fluorescence Imaging of Pancreatic Cancer Using a Fluorescently Labelled Anti-CEA Nanobody Probe: A Preclinical Study
Source: Biomolecules. 2023 Mar 30;13(4):618. doi: 10.3390/biom13040618 (PMC10135789; doi:10.3390/biom13040618)

Near-infrared fluorescence imaging of pancreatic cancer using a fluorescently labelled anti-CEA nanobody probe: a preclinical study.

Van Manen et al.

Supplementary data

Figure S1

Overview on flowcytometry results.

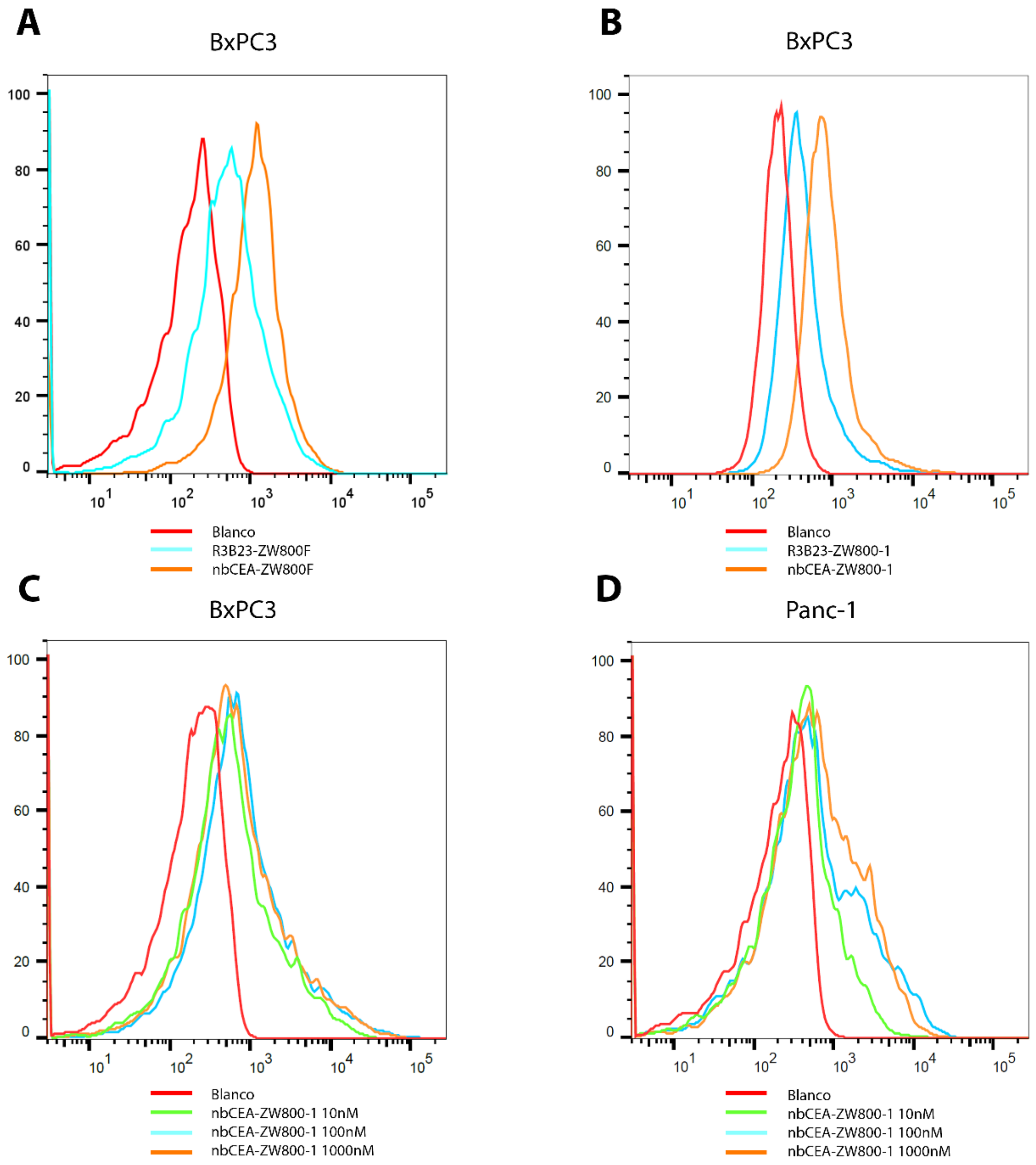

Figure S2

Biodistribution data of NbCEA5-ZW800-1 in the pancreatic orthotopic models (N=2). Scale bar is 10 mm. *Abbreviations: Lu=lungs; Ht=heart; Li=liver; Tu=tumor; Pa=pancreas; Sp=spleen; Int=small intestines; St=stomach; Ki=kidneys; Co=colon; Sk=skin; Mu=muscle.*

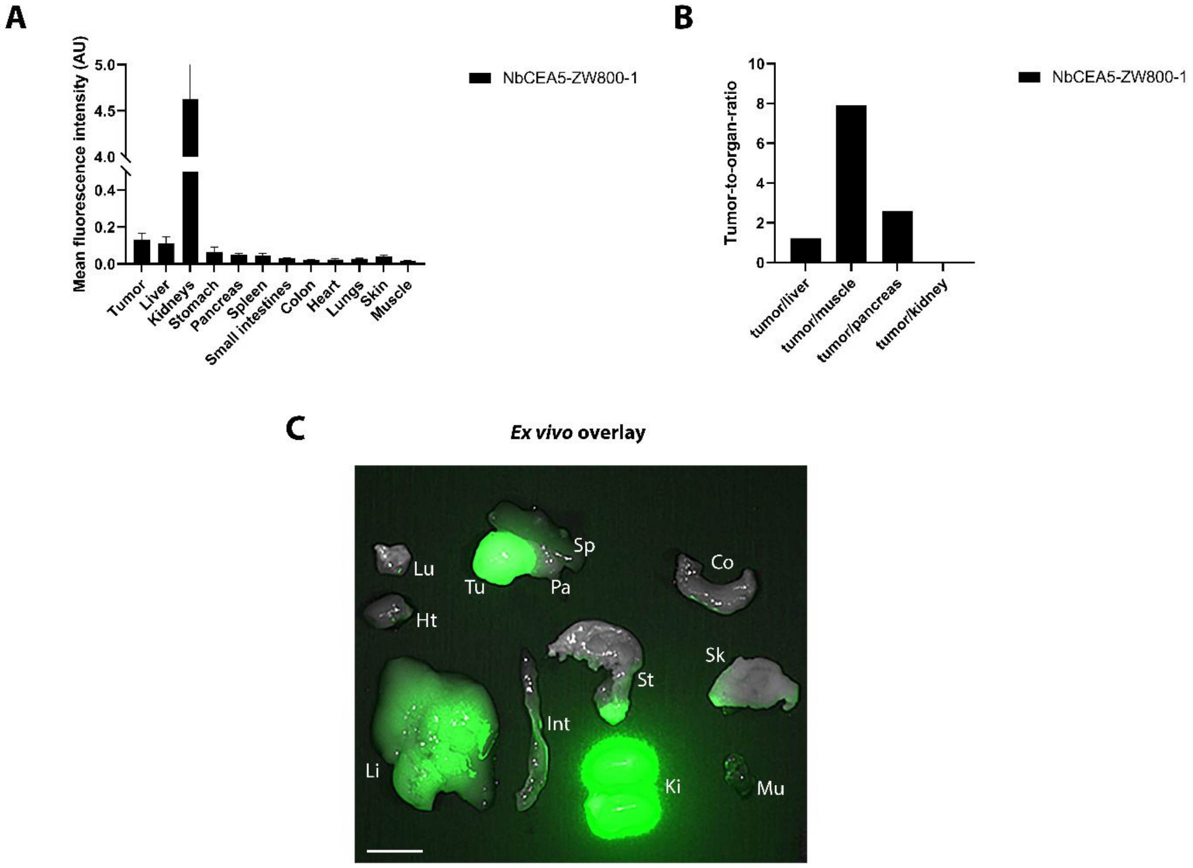

Supplement: Supplementary file 1 [file biomolecules-13-00618-s001.zip › biomolecules-2269041-supplementary.pdf]
